# Application of fatty acid-based eutectic mixture as a phase change material in microencapsulation of drugs: preparation, characterization and release behavior

**DOI:** 10.1186/s13065-025-01406-4

**Published:** 2025-02-17

**Authors:** Fariba Ghaffari, Hemayat Shekaari

**Affiliations:** Department of Physical Chemistry, University of Tabriz, Tabriz, Iran

**Keywords:** Microencapsulation, Phase change material, Eutectic mixture, Drug, Heat capacity

## Abstract

Recently, microencapsulation has developed in various industries with its versatile applications. Its profound impact is particularly notable in the chemical, food, and pharmaceutical sectors. Among its research areas, the microencapsulation of drugs using phase change materials (PCMs) stands out as a groundbreaking advancement in drug delivery systems. This innovative approach involves encasing drugs within a PCM shell, significantly enhancing their stability and delivery regulation. The focus of our study is the microencapsulation of certain drugs with poor water solubility namely, cyclosporine, baclofen, and biotin within a bio-based PCM. It has identified PCMs with phase transition temperatures near human body temperature ( 310 K) as ideal candidates for this purpose. A eutectic mixture of stearic-lauric acid in a 1:3 mole ratio was selected for its optimal phase change properties to create microcapsules with core–shell morphology in spherical form. Our comprehensive characterization of the microcapsules, validated by FT-IR and SEM techniques, confirms their proper formation. All studied drugs microencapsulated with the PCM exhibited an excellent thermal stability at working temperature from thermal stability analysis based on TGA results. Furthermore, differential scanning calorimetry (DSC) tests conducted on the microencapsulated drugs obtained the melting point of all three microencapsulated drugs near the melting point of PCM. Also, the release behavior of drugs from drug delivery method was investigated in PBS (pH 7.4) and two temperatures (310.15 and 318.15) K. Drug release occurred sustainably, such that 50% and about 60% of the total of each drug was released from the microcapsules at mentioned temperatures respectively during 24 h.

## Introduction

Microncapsulation, a technique involving the containment of active compounds within a specific structure, finds widespread application in the pharmaceutical industry [1, 2]. Many drugs and vitamins exhibit poor stability and short half-lives, necessitating frequent administration. For instance, biotin and baclofen, with half-lives of only 2–6 h, require frequent dosing for optimal therapeutic effects [3, 4]. Microencapsulation, a sophisticated approach, involves enclosing drugs within a protective coating to form microcapsules. This technique offers numerous advantages, including enhanced bioavailability, extended shelf life, and improved stability for sensitive compounds like vitamins, antibiotics, and enzymes [5, 6]. By controlling the release rate of the active ingredient, microencapsulation can minimize drug reactivity, reduce toxicity, and even mask unpleasant tastes. This technology plays a pivotal role in the development of advanced drug delivery systems, enabling targeted and controlled release for optimized therapeutic outcomes [7–17]. Various methods are employed for drug microencapsulation [18–20], including emulsification. This technique involves dispersing two immiscible liquids to create a stable mixture, such as an oil-in-water emulsion where oil droplets are dispersed in water [18]. Common encapsulating materials include biopolymers and lipids.

One promising area of research in drug delivery utilizes phase change materials (PCMs) for microencapsulation [21–28]. Ideally, stimuli-responsive materials for clinical translation should possess several key characteristics: The payloads should be gated to prevent prerelease without causing toxicity to the body; they should also respond quickly and reversibly to a specific stimulus, allowing for repeated activation and deactivation of the release; they should be able to be loaded at high loading capacities with a variety of payload types; they should be biocompatible and biodegradable to ensure that the carrier materials are safe and can be broken down or removed from the body after use; and finally, they should be easily obtained and engineered. While polymeric materials have been extensively researched for controlled release [29–31], challenges such as complex preparation, limited degradability, and potential toxicity hinder their clinical translation. PCMs offer a novel approach to controlled release [32]. These materials undergo a solid–liquid phase transition, absorbing and releasing heat at a nearly constant temperature [33, 34]. This phase change not only involves energy storage but also significantly alters the material's physicochemical properties, including density and molecular mobility. Temperature-responsive release systems can be established by controlling the release of chemicals that are trapped within a solid matrix through the utilization of enhanced mobility during a solid-to-liquid phase transition [35–39].

Fatty acid-based PCMs receive special attention within the PCM spectrum because of their abundance in nature, low toxicity, biodegradability, versatility, vast availability, and affordability. For example, recent studies have shown the effectiveness of fatty acids in achieving stable encapsulation and controlled release of bioactive compounds [32, 40–43]. Because of their excellent biocompatibility, natural fatty acids are a good choice for controlled release; nevertheless, it can be difficult to achieve a PCM that is exclusively made of a pure fatty acid with a melting point that is close to the body's physiological temperature (310.15 K). Additionally, a single fatty acid has a tendency to solidify into a highly crystalline structure, which causes compounds to separate from the fatty acid matrix and reduces the drug's ability to be encapsulated while causing undesirable fast releases [44–46]. In order to overcome these challenges, the idea of a eutectic blend that consists of two or more fatty acids and has a lower melting point than any one of the constituents stands out as a workable substitute for single-component fatty acids in terms of reaching the appropriate melting points and modifying the crystallization behavior of fatty acids in order to enhance drug loading capacity [38, 47–54].

Therefore, a mixture of stearic acid and lauric acid as bio-based PCM with mole ratio of 1:3 was chosen [55] to microencapsulate three poor soluble drugs in water: cyclosporine, baclofen, and biotin with oil-in-water emulsions method, where the drug (usually hydrophobic) is dissolved or dispersed in the oil phase. The properties of microencapsulated drugs were analyzed through differential scanning calorimetry (DSC). Morphology and particle size distribution (PSD) were observed using scanning electron microscope (SEM). The shell formation around the drugs core was confirmed by Fourier transform infrared spectroscopy (FT-IR). Also, thermal degradation temperatures were examined by thermogravimetric analysis (TGA). Finally, the release behavior of each microencapsulated drug was investigated from the drug delivery method.

## Experimental measurements

### Chemicals

An eutectic mixture made by stearic acid and lauric acid in a (1:3) mole ratio was used as a sell for some widely used drugs. The chosen drugs as the core material were biotin, baclofen, and cyclosporine. The surfactant in this work was sodium laurylsulfonate (SLS). Polyvinyl alcohol (PVA) and polyvinylpyrrolidone (PVP) were used as stabilizers. The details of used chemicals such as purity, CAS number, and origin are mentioned in Table 1.

**Table 1:** Information of used chemicals

Chemicals	Source	CAS No.	Mass percent (purity)
Biotin	Zahravi (Iran)	58-85-5	≥ 99
Baclofen	Zahravi (Iran)	1134-47-0	≥ 99
Cyclosporine	Zahravi (Iran)	59865-13-3	≥ 99
Polyvinylpyrrolidone (PVP)	Milipore	9003-39-8	≥ 99
Polyvinyl alcohol (PVA)	Sigma-Aldrich	25213-24-5	≥ 99
Sodium laurylsulfonate (SLS)	Sigma-Aldrich	151-21-3	≥ 99
Lauric acid	Sigma-Aldrich	143-07-7	≥ 99
Stearic acid	Sigma-Aldrich	57-11-4	≥ 97
Ethanol	Merck	64-17-5	> 0.99
PBS	Merck	10010023	–
Dialysis sacks	Merck	–	–

The suppliers were provided the purities of the used components

### Fourier transform-infrared spectroscope (FT-IR) spectra

The Fourier transformation infrared spectroscope (FT-IR) spectra of the microencapsulated drugs has been recorded using a Bruker Tensor 270-KBr through the KBr pellet technique.

### Scanning electron microscope (SEM)

The detailed examination of the prepared microcapsules was conducted using a high-precision instrument known as a scanning electronic microscope (TESCAN MIRA3 FEG-SEM) to assess their morphology and microstructure. The advanced capabilities of this microscope allowed for an in-depth visualization of the microcapsules’ surface characteristics and internal composition, providing valuable insights into their structural integrity and potential applications.

### Thermogravimetry analysis (TGA)

In order to assess the thermal stability of the produced microcapsules across a range of (323.15–973.15) K under N2 (30 mL·min^−1^), thermogravimetry analysis was performed on the samples using a thermal analysis apparatus (METTLER TOLEDO, TGASDTA 851e).

### Differential scanning calorimetry (DSC)

Differential scanning calorimeter (Netzsch DSC-200 F3) was used to determine the thermophysical properties of synthesized encapsulated drugs. The heat of fusion and heat capacities were calculated from this method. To achieve this goal, three thermal phases were performed with cooling and heating rates of 10 K/min. To remove any thermal history the samples were first cooled to 203 K, and then the temperature was kept at same degree for 10 min. The encapsulated drugs were then heated to 373 K. From the heating stage, it was possible to determine the thermal properties of the encapsulated drugs.

The microcapsules specific heat capacities were measured for the temperature range of (298.15–328.15) K at 10 K/min. The samples of microcapsules were weighed using an electronic balance with an uncertainity of ± 10^−4^ g.

### Preparation of the microcapsules

Biotin, baclofen, and cyclosporine were chosen as core material in a PCM as the microcapsules shell. An eutectic mixture containing stearic acid and lauric acid with (1:3) mole ratio was used as phase change material [37]. In the synthesis process a 500 ml three-neck flask, an oil thermostat bath and digital stirring rate control were used. A Rushton turbine type stirrer with six vertical blades was used in this work. For the synthesis of microcapsules first two phases were prepared separately, aqueous phase consist of PVP and PVA as stabilizer, SLS as emulsifier, and demineralized water (DMW), oil phase consist of (stearic-lauric) acid eutectic mixture and drug (biotin, baclofen, or cyclosporine). Stabilizers help maintain the uniformity of emulsions by preventing the separation of phases. This is particularly important during the formation of microcapsules, where the drug is often dispersed in an oil or water phase. Stabilizers also, contribute to maintaining a consistent droplet size in the emulsion, which is critical for producing microcapsules of uniform size. The simultaneous use of two stabilizers, PVP and PVA, in the encapsulation of drugs is often employed to take advantage of their complementary properties; PVP is a well-known stabilizer that provides excellent colloidal stability. It can adsorb onto the surface of drug particles, reducing the interfacial tension and preventing the coalescence of the emulsion droplets, PVA, on the other hand, is a good stabilizer for aqueous dispersions and provides strong film-forming properties. It can create a protective barrier around the droplets, further enhancing the stability of the emulsion. The combination of PVP and PVA can provide a more robust stabilization of the emulsion, ensuring uniform droplet size and preventing phase separation, which is critical for the formation of uniform microcapsules. Also, emulsifiers play a critical role in the microencapsulation of drugs, a technique used to enhance the stability, control the release, and improve the bioavailability of pharmaceuticals. In microencapsulation of drugs, emulsifiers stabilize oil-in-water emulsions, the emulsifier reduces the interfacial tension between the oil and water phases, preventing coalescence and ensuring a stable emulsion [56]. This oil phase was then merged with the aqueous phase in the flask, under intense stirring. The mixture was agitated for an hour at a constant temperature of 333.15 K. After that, temperature by the rate of 1 K/min was gradually decreased (< *T* = 293.15 K) [56]. Whatman filter paper No. 41, was used to filter the prepared suspension, then the filter was dried at 298.15 K in a Memmert VO500 PMP500 vacuum oven. Table 2 displays the ideal microencapsulation synthesis recipe.

**Table 2:** The recipe for microencapsulation of biotin, baclofen, and cyclosporine in stearic-lauric acid shell

	Aqueous phase				Oil phase	
	DMW (g)	SLS (g)	PVA (g)	PVP (g)	PCM (g)	Drug (g)
Biotin	50	0.002	0.231	0.221	5.0	1.5
Baclofen	50	0.002	0.231	0.221	4.0	1.0
Cyclosporine	50	0.002	0.231	0.221	5.0	2.0

### Drug release study

To evaluate the release behavior of the studied drugs, we incubated encapsulated drugs in PBS (pH 7.4) 310.15 K and 318.15 K in a dialysis bag (molecular mass cutoff 12,000 Da) and next immersed this bag into 25 mL of PBS.

A predetermined amount of these particles (100 mg) containing the drug was dispersed in 3 ml of phosphate buffer with a pH of 7.4. Since the free drug is not soluble in water, the solution was subjected to centrifugation at 3000 rpm for 10 min at specific time intervals to separate the released drug, which formed a pellet, from the particles that still contained drug. The released drug was then dissolved again in a 3 ml ethanol solution, consisting of a volume ratio of 2:8 for water and ethanol, respectively. The absorbance of this solution was measured using a spectrophotometer at a wavelength of 428 nm. To determine the concentration of the released drug, a standard curve of the drug in ethanol was employed. Finally, the percentage of drugs released was calculated using a specific equation [57]:

1 $$Release(\% ) = \frac{{Drug_{{_{rel} }} }}{{Drug_{tot} }} \times 100$$

where, *Drug*_*tot*_ is the total amount of encapsulated drug in the stearic-lauric acid eutectic mixture and *Drug*_*rel*_ is the concentration of released drug measured at time *t*.

## Results and discussion

### FT-IR results

The initial Fourier transform infrared spectroscopy (FT-IR) analysis of the fabricated microcapsules was conducted to ascertain the presence of phase change material (PCM) within the shell composition. The FT-IR spectrum serves as a diagnostic tool to verify the encapsulation of the core substances, which include biotin, baclofen, and cyclosporine, characterized by their distinct conjugated bonds, aromatic structures, and cyclohexane rings.

**Fig. 1:**
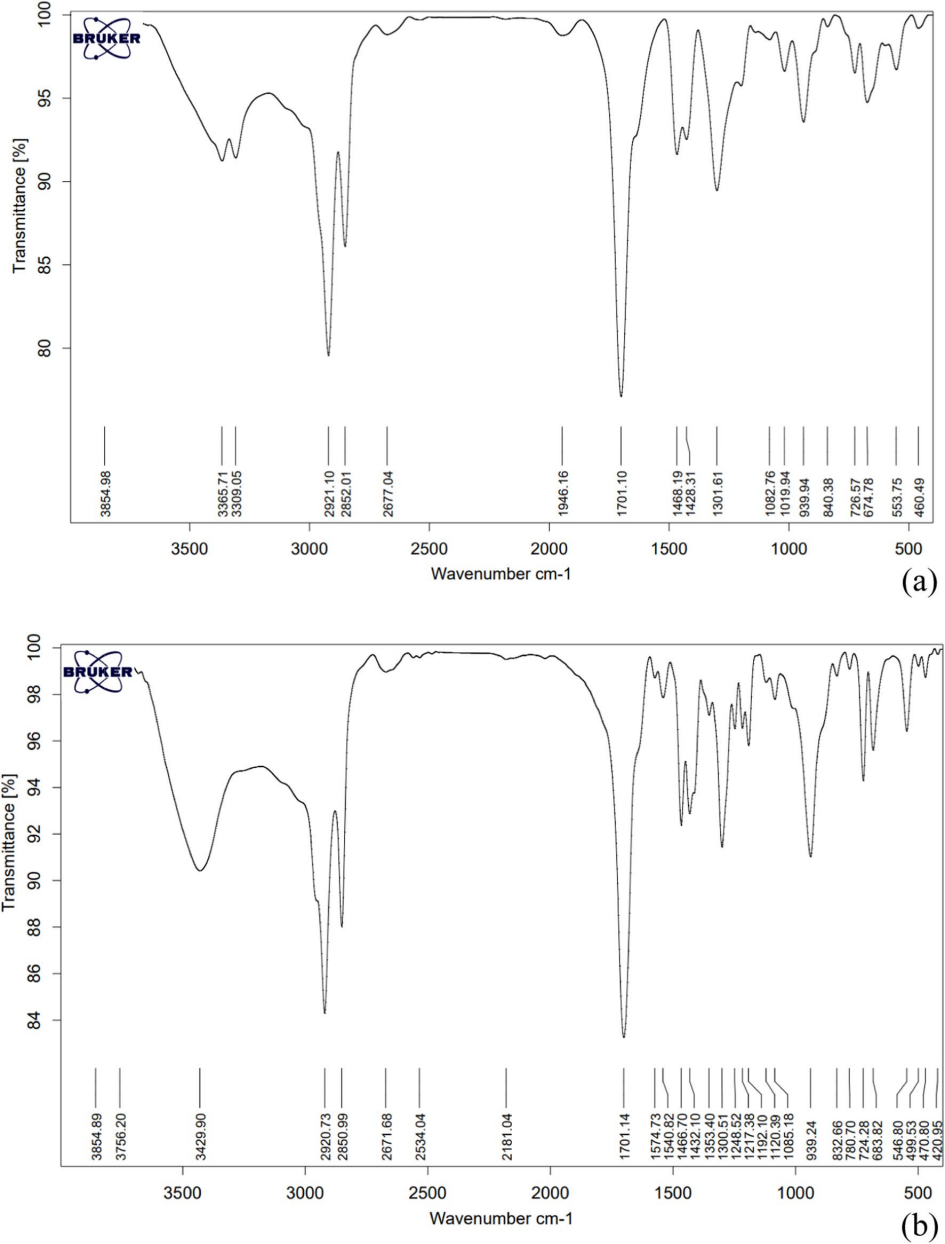

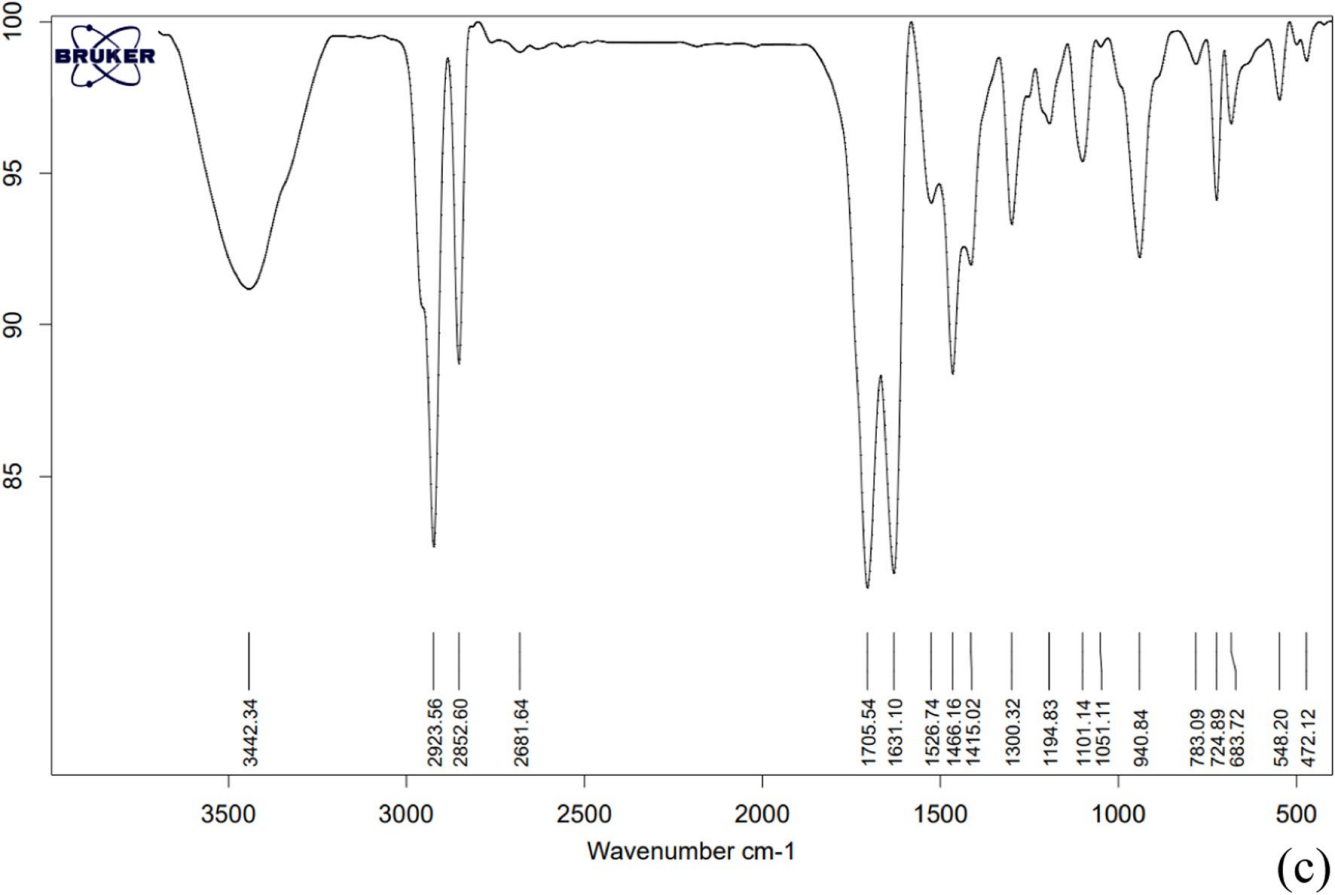
The FT-IR spectrum of the; **a** encapsulated biotin, **b** encapsulated baclofen, **c** encapsulated cyclosporine

Figure 1 presents the FT-IR spectra of the synthesized microcapsules, revealing characteristic absorption peaks. To validate the successful encapsulation of mentioned drugs within the stearic-lauric acid eutectic mixture, the FT-IR spectra of the encapsulated drugs were compared with the reported spectra of free drugs available in the literature [58–60]. Figure 1a, shows biotin microcapsules FT-IR spectra, biotin has an imidazolidone ring, with C = O stretching around 1700 cm⁻^1^, N–H stretching (Amide Group) between 3300 and 3500 cm⁻^1^, and C-N Stretching (Secondary Amine) between 1270 and 1350 cm⁻^1^ [58]. The stretching peak of C = O appears at around 1701 cm⁻^1^ but with reduced intensity, indicating interaction with the stearic-lauric acid shell. N–H stretching in the 3300–3500 cm⁻^1^ range shows a shift to lower wavenumbers, with reduced intensity, suggesting hydrogen bonding between the biotin core and the shell. Also, C-H stretching peaks of the shell material (2921 and 2852 cm⁻^1^) are present, confirming microencapsulation. Shifts and reductions in the characteristic peaks of biotin (e.g., N–H and C = O) compared to free biotin in the literature confirm encapsulation.

Figure 1b, shows baclofen microcapsules FT-IR spectra, baclofen contains a carboxylic acid group (C = O stretching near 1700 cm⁻^1^), N–H Stretching relating to amine group between 3300 and 3500 cm⁻^1^, and C-H stretching between 2800 and 3000 cm⁻^1^ [59]. The C = O stretching peak appears at ~ 1701 cm⁻^1^ with slight broadening, suggesting interaction with the shell. Stretching of N–H in the 3300–3500 cm⁻^1^ range is reduced and slightly shifted, indicative of hydrogen bonding or encapsulation effects. Also, shell material’s C-H peaks (2920 and 2850 cm⁻^1^) dominate the spectrum. Figure 1c, shows cyclosporine microcapsules FT-IR spectra, cyclosporine is a cyclic peptide with amide groups (C = O stretching near 1650 cm⁻^1^), N–H stretching (amide group) between 3300 and 3500 cm⁻^1^, and C-N stretching between 1200 and 1400 cm⁻^1^ [60]. The C = O stretching peak appears at 1705 cm⁻^1^ and 1631 cm⁻^1^, with slight shifts compared to the free drug, indicating interaction with the shell. Reduced intensity of N–H stretching in the 3300–3500 cm⁻^1^ range, suggesting hydrogen bonding between the drug and the shell material. Likewise, peaks at 2923 and 2852 cm⁻^1^ related to C-H (shell material) stretching are distinct, confirming the presence of the shell. The FT-IR spectral analysis provides compelling evidence of successful microencapsulation of biotin, baclofen, and cyclosporine drugs within the stearic-lauric acid shell (PCM). Characteristic peaks of the free drugs were observed with significant shifts and intensity changes, confirming interactions with the shell material. The presence of shell-specific peaks further validates the formation of a robust core–shell structure, establishing the efficacy of the microencapsulation process.

### SEM results

**Fig. 2:**
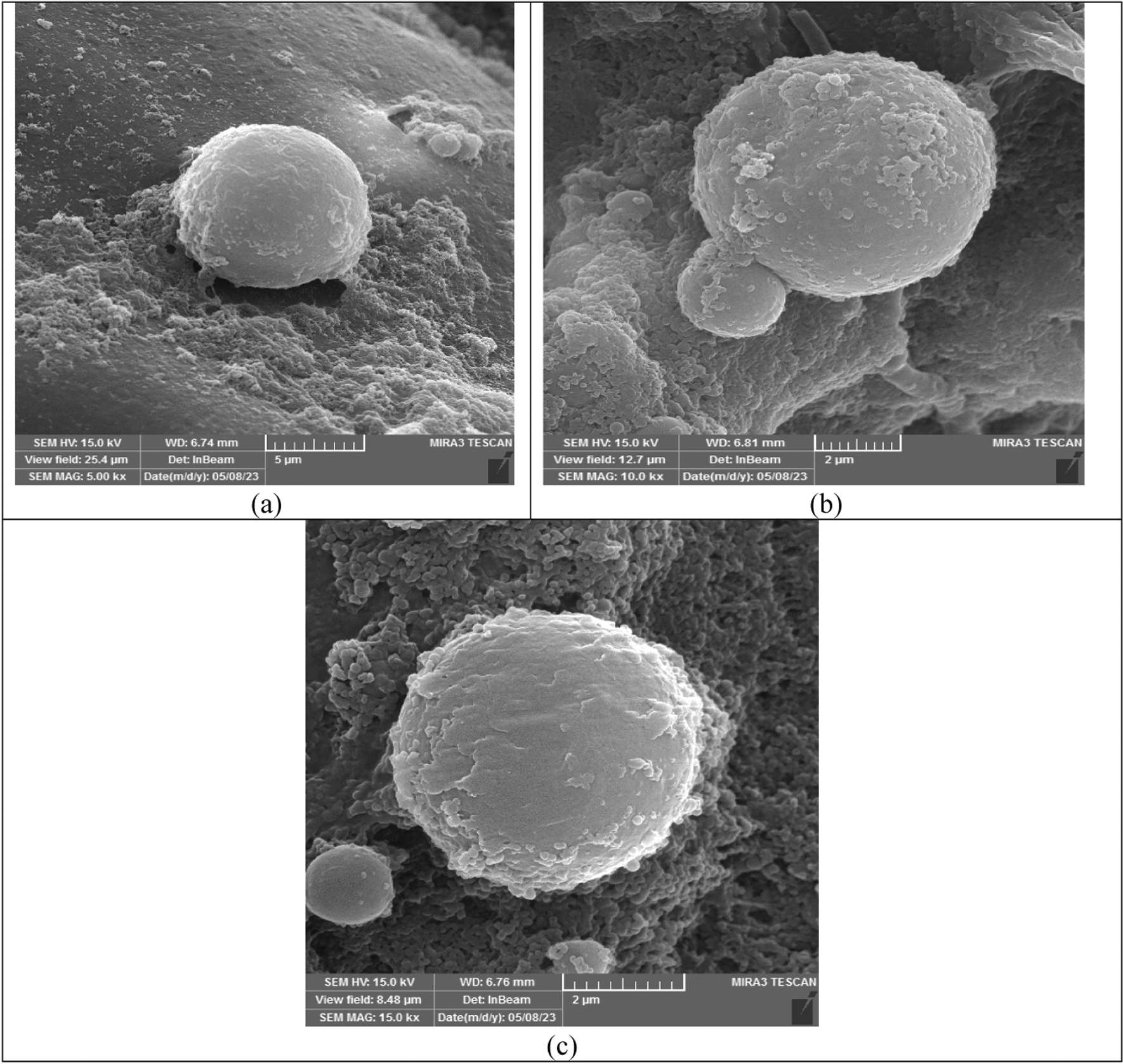
The SEM images of the; **a** microencapsulated biotin, **b** microencapsulated baclofen, **c** microencapsulated cyclosporine

Scanning Electron Microscopy (SEM) serves as an alternative technique for evaluating the structure and morphology of microcapsules. In Fig. 2, the SEM results for the encapsulated drugs are displayed. Free biotin typically appears as irregularly shaped crystals with sharp edges, baclofen appears as fine, needle-like crystals, also, free cyclosporine often exhibits an amorphous appearance, with particles lacking a defined shape or uniformity. The analysis of Fig. 2 clearly demonstrates significant morphological changes in all three drugs upon encapsulation. The transition from irregular or crystalline forms to spherical microcapsules validates the encapsulation process. This Figure shows that the formed microcapsules are acceptable in terms of morphology and sphericity. Furthermore, the microcapsules fall within the size range of 2–5 μm. However, there are problems in terms of the uniformity of the surface of the capsules, which may be due to the presence of impurities and initial materials in the final product. Different factors such as the rate of cooling during microcapsule formation, the interfacial tension between the drug and PCM, and the concentration and type of emulsifier have a significant effect on the morphology of microcapsules. A faster or slower cooling rate could lead to different crystallization patterns or structures in the encapsulating material. Also, an increased emulsifier concentration generally leads to smoother and more consistent surfaces because the emulsifier reduces the surface tension between the core and the surrounding medium, allowing for better encapsulation. Based on this, by changing the concentration and type of emulsifier, microcapsules with controlled structures can be obtained [61]. In this study, the non-uniformity of the capsule surface can be due to these two reasons.

The SEM findings provide further substantiation of the successful microencapsulation process. They validate that the selected parameters, including the shell-to-core ratio, stabilizers, emulsifier, impeller, stirring rate, and temperature program, were appropriately employed to encapsulate the investigated drugs within a PCM shell comprising a mixture of stearic acid and lauric acid in a 1:3 mole ratio.

### TGA results

**Fig. 3:**
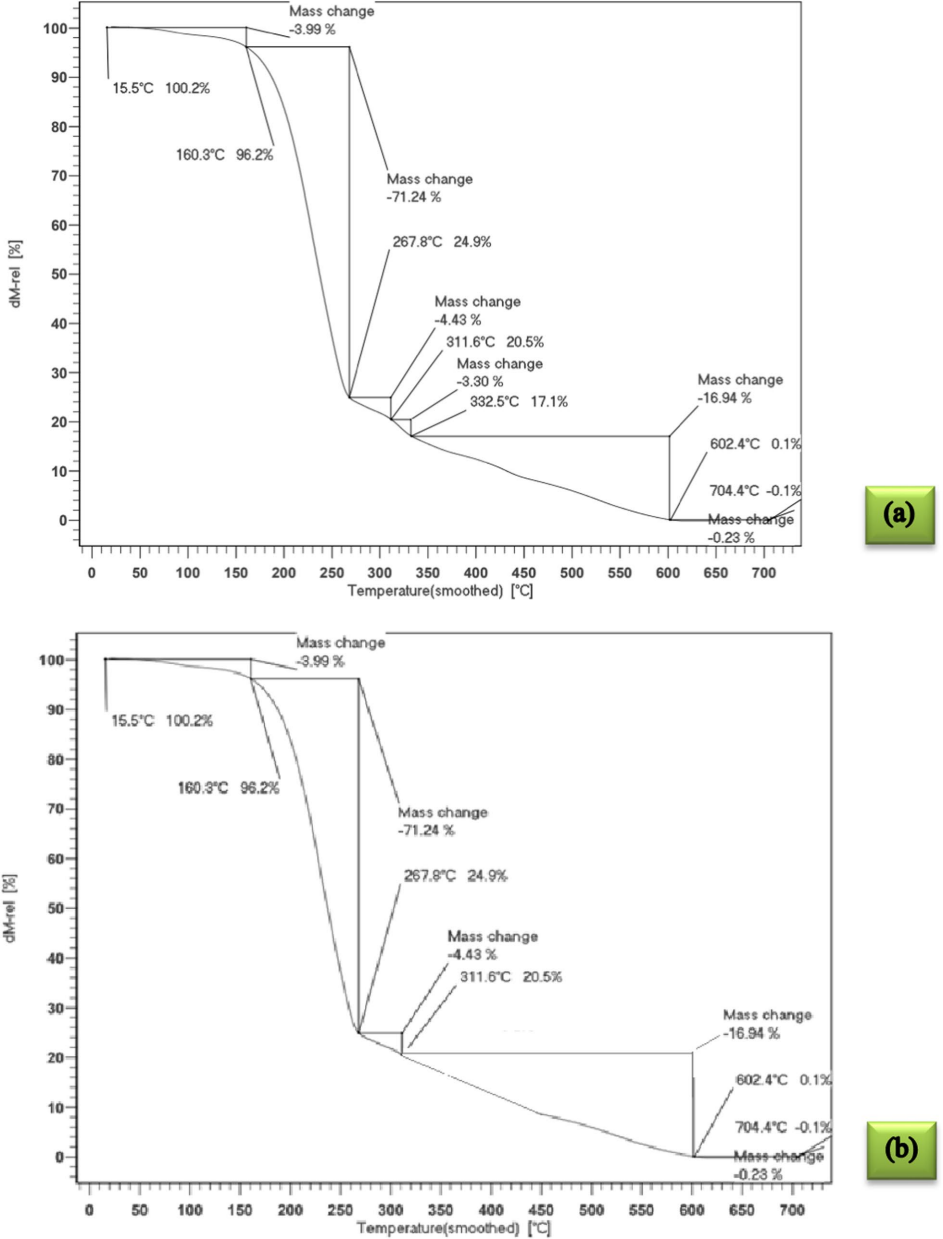

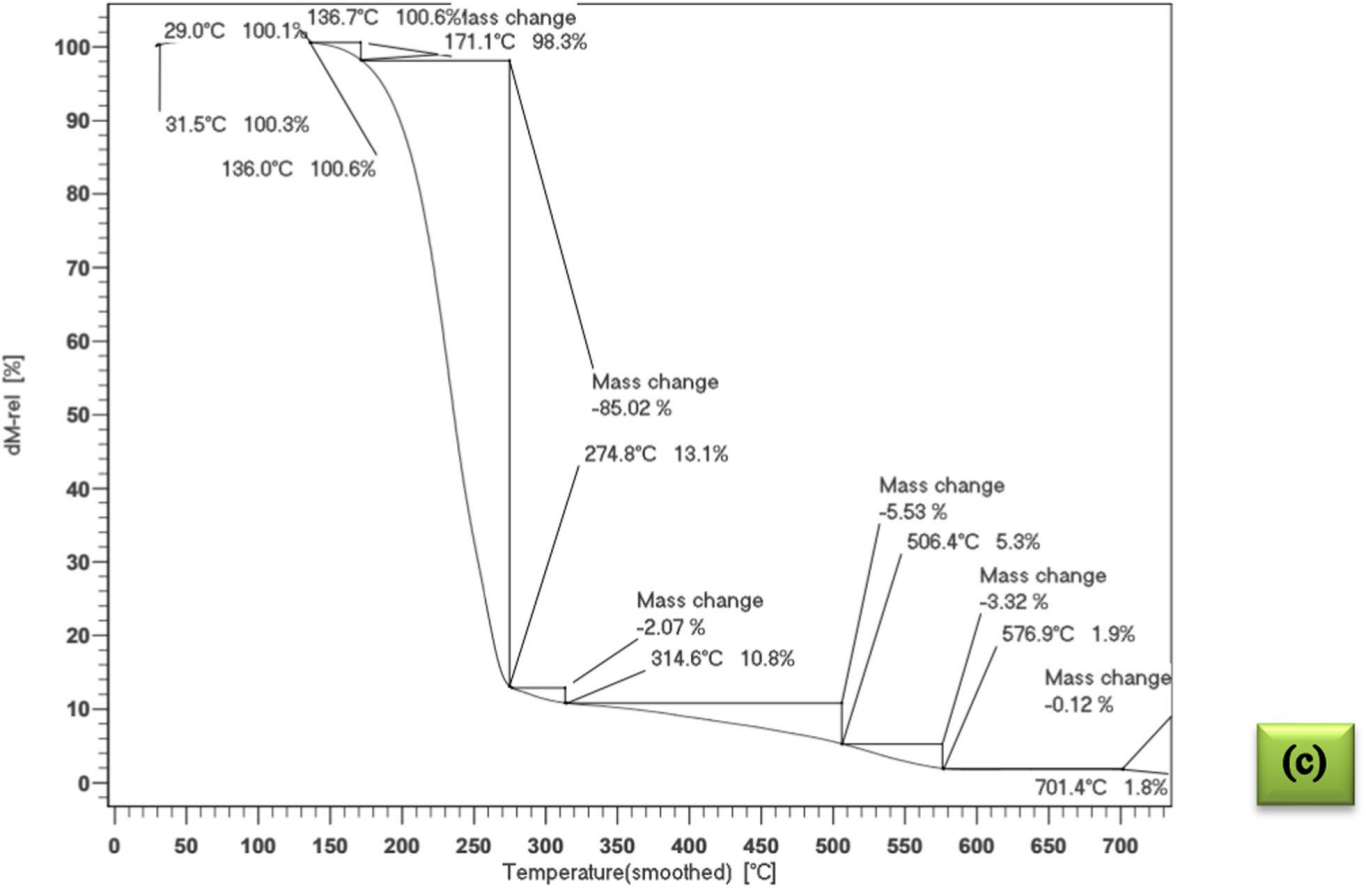
The quasistatic TGA of the; **a** encapsulated biotin, **b** encapsulated baclofen, **c** encapsulated cyclosporine

The thermal behavior of the microencapsulated drugs was analyzed through thermogravimetric analysis (TGA) curves, as illustrated in Fig. 3. Additionally, Table 3 presents the charred residue content at 973.15 K and the corresponding temperatures at which the greatest weight loss occurs. Figure 3 also demonstrates the two-step thermal degradation mechanisms. The initial step, depicted in Fig. 3, involves the release of water molecules adsorbed within the microcapsule network and occurs within the temperature range of (293.15 to 423.15) K. Since many drugs exhibit low stability at ambient temperatures, microencapsulation can significantly enhance their stability. The synthesized microcapsules exhibit approximately 90% thermal stability up to 423.15 K, making them a suitable choice for various applications.

**Table 3:** The TGA and DSC data of microencapsulated biotin, baclofen and cyclosporine drugs by stearic-lauric acid PCM

Chemicals	Melting point (K)	Latent heat (kJ·kg^−1^)	Thermal stability or residue amount (%) up to 150 °C
Microencapsulated biotin	319.65	129	96
Microencapsulated baclofen	317.65	55	89
Microencapsulated cyclosporine	317.95	98	98

There is another weight loss between *T* = (441.15–547.15) K, which shows the decomposition of the core material. After this stage, the thermal decomposition of the PCM (stearic-lauric acid) shell continued up to about *T* = 591.15 K.

### DSC results

**Fig. 4:**
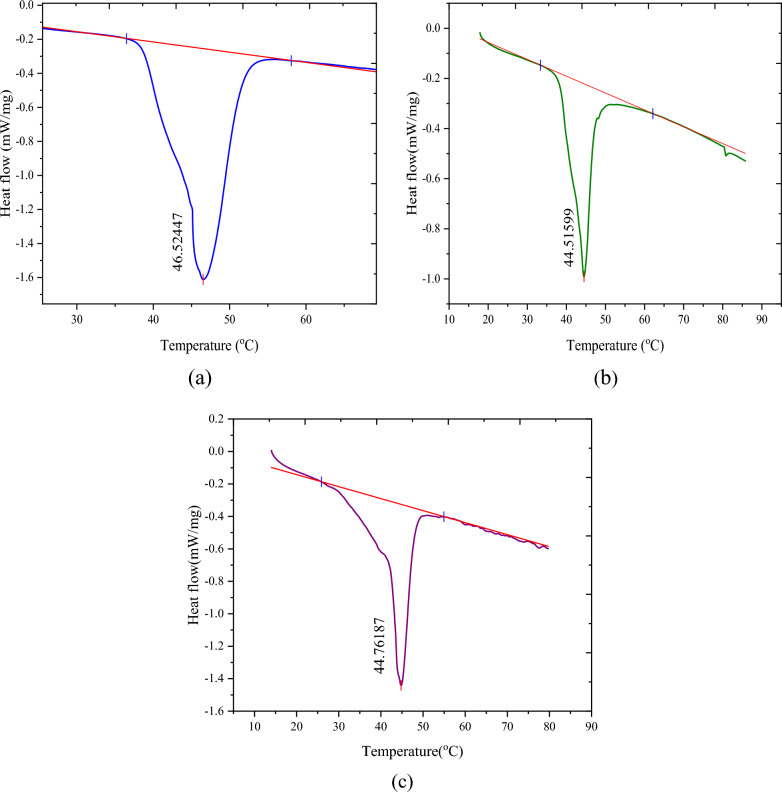
The DSC analysis of the drugs; **a** microencapsulated biotin, **b** microencapsulated baclofen, **c** microencapsulated cyclosporine

**Fig. 5:**
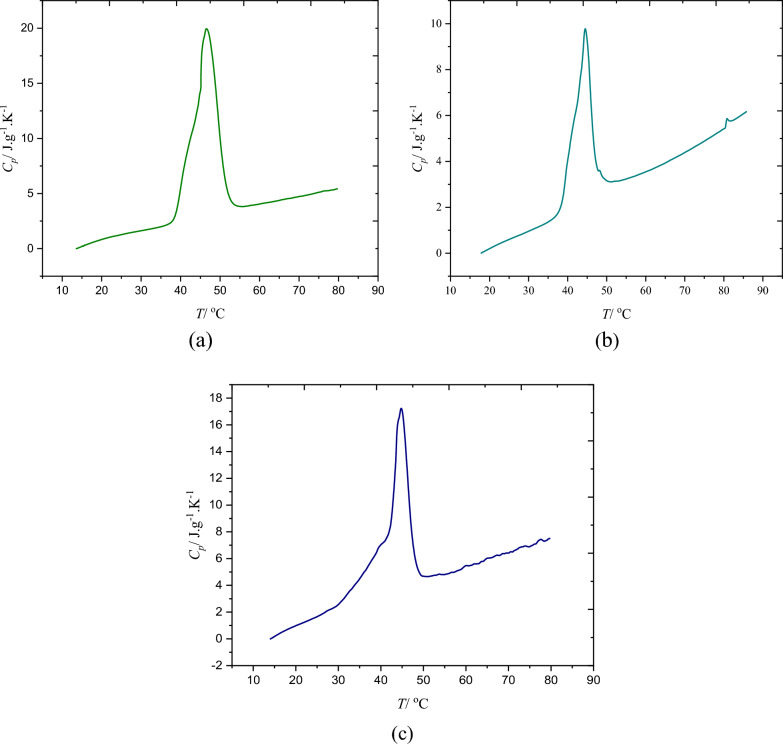
The heat capacity of the; **a** microencapsulated biotin, **b** microencapsulated baclofen, **c** microencapsulated cyclosporine

The experimental findings presented in Fig. 4 and Table 3 provide insights into the calorimetry results for a series of the microencapsulated drugs. Figure 4 displays the exothermic heating curves, obtained through DSC, for biotin, baclofen, and cyclosporine. The corresponding melting points for these drugs are recorded as 319.67 K, 317.66 K, and 317.91 K, respectively, as listed in Table 3. These results suggest that the encapsulated drugs possess similar structural characteristics, thus requiring comparable amounts of energy for the melting process. Notably, the phase transition properties of the encapsulated drugs exhibit a striking similarity to the structure of fatty acids. From DSC results, the values of melting latent heat were calculated for encapsulated drugs as 129.00, 95.00, and 98.00 kJ·kg^−1^ for biotin, baclofen, and cyclosporine, respectively, which are shown in Table 2. Furthermore, the DSC results were utilized to determine the specific heat capacity (C_p_) values of the encapsulated drugs at various temperatures, as depicted in Fig. 5. Although all the encapsulated drugs share stearic-lauric acid PCM as a common component, there are differences in the heat capacity values among the studied drugs due to variations in their respective cores. Significantly, the C_p_ values for biotin surpass those of the other two encapsulated drugs.

Overall, the obtained results shed light on the calorimetry analysis of microencapsulated drugs. They highlight the similar structural characteristics and energy requirements for melting among the studied drugs, with the phase transition properties closely resembling fatty acids. The observed differences in heat capacity values are attributed to variations in the drug cores, underscoring the influence of core composition on the thermal behavior of the encapsulated drugs.

### Drug release study

In this study, it has been investigated the release of some poor soluble drugs namely biotin, baclofen, and cyclosporine from encapsulation drug delivery method. To evaluate the release of each drug, we conducted experiments and observed that drugs release occurred prior to the 24-h interval.

**Fig. 6:**
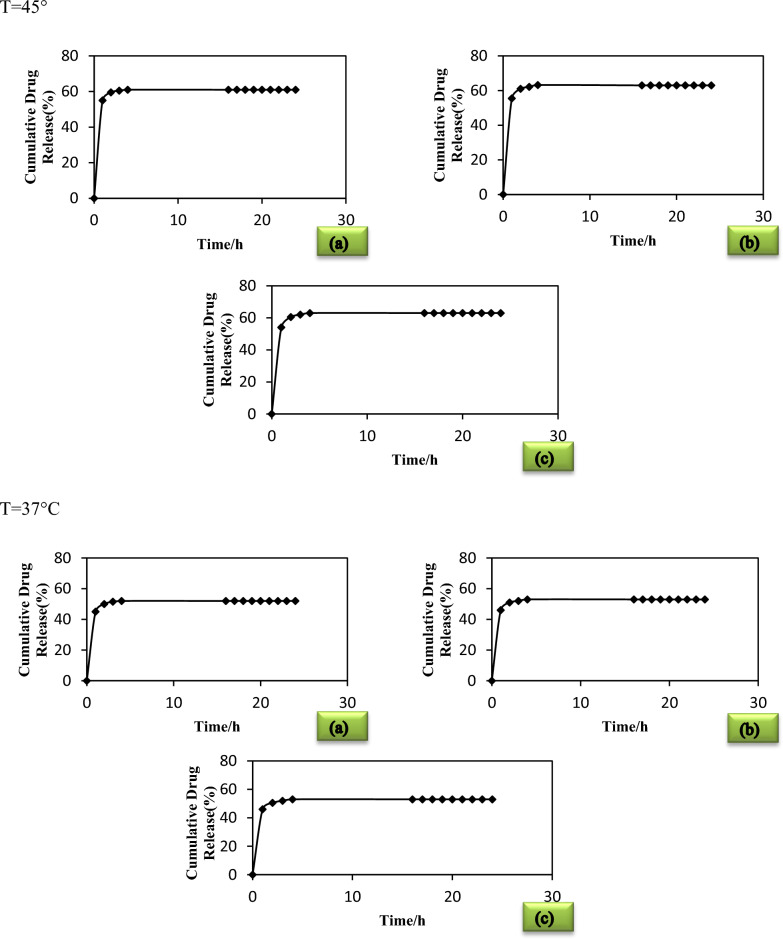
The release kinetics of microencapsulated drugs demonstrates ~ 50% and about 60% release of drugs from the drug-encapsulated at 24 h when dispersed in phosphate based buffer at physiological pH (7.4). **a** Biotin microcapsule; **b** Baclofen microcapsule; **c** Cyclosporine microcapsule

The in release profile of the each microencapsulated drug by fatty acid-based eutectic mixture at physiological pH (7.4) is illustrated in Fig. 6. Drug release occurs sustainably, such that 50% and about 60% of the total drug is released from the microcapsules at 310.15 and 318.15 K respectively during mentioned time. The evaluated data is demonstrative of the temperature effect on the drug release which indicated a high amount of drug release at 318.15 K rather than 310.15 K.

## Conclusions

In this study, a bio-based phase change material composed of stearic acid and lauric acid with a mole ratio (1:3) was used to microencapsulate three low water-soluble drugs namely: biotin, baclofen, and cyclosporine. Characterization of the produced microcapsules was confirmed by FT-IR, and SEM techniques. Thermal stability analyzes (TGA) show that the thermal stability of biotin, baclofen, and cyclosporine is 92, 89, and 98%, respectively which indicates the high thermal stability of the encapsulated drugs. Finally, the DSC test was done for microcapsulated drugs. The temperature at which the drugs enclosed within the capsules undergo a phase change from solid to liquid was measured and found to fall within the range of 317.15–319.15 K. The amount of heat required to accomplish this phase transition, known as the latent heat of fusion, was determined to be 129.00, 95.00, and 98.00 J·g^−1^·K^−1^ for biotin, baclofen, and cyclosporine, respectively. Furthermore, the release data shows the effect of temperature on the drug release. These results indicated a high amount of drug release at 318.15 K compared to 310.15 K.

## Data Availability

All data generated or analysed during this study are included in this article.
